# Protective roles of autophagy in retinal pigment epithelium under high glucose condition via regulating PINK1/Parkin pathway and BNIP3L

**DOI:** 10.1186/s40659-018-0169-4

**Published:** 2018-07-16

**Authors:** Chengchi Huang, Hong Lu, Junyu Xu, Hongmin Yu, Xiaodan Wang, Xiaomei Zhang

**Affiliations:** 1 Ophthalmology Hospital, The First Affiliated Hospital of Harbin Medical University, 23 Youzheng Street, Nangang District, Harbin, 150001 China; 2 Department of Ophthalmology, The Fourth Affiliated Hospital, Harbin Medical University, 17 Yiyuan Street, Nangang District, Harbin, 150001 China

**Keywords:** Diabetic retinopathy, Retinal pigment epithelium, Autophagy, High glucose

## Abstract

**Background:**

Our study aimed to investigate the roles of autophagy against high glucose induced response in retinal pigment epithelium (ARPE-19 cells).

**Methods:**

The morphological changes and reactive oxygen species (ROS) generation in ARPE-19 cells under high glucose treatment were respectively detected using the transmission electron microscopy and flow cytometry. The expression levels of Parkin, PINK1, BNIP3L, LC3-I and LC3-II in ARPE-19 cells received high glucose treatment were measured by western blot after pretreatment of carbonyl cyanide m-chlorophenylhydrazone (CCCP), 3-methyladenine (3-MA), *N*-acetyl cysteine (NAC) or cyclosporin A (CsA) followed by high glucose treatment.

**Results:**

ARPE-19 cells subjected to high glucose stress showed an obvious reduction in the LC3-I expression and significant increase in the number of autophagosomes, in the intracellular ROS level, and in the expression levels of Parkin, PINK1, BNIP3L and LC3-II (p < 0.05). Pretreatment with CCCP significantly reduced the LC3-I expression and increased the expression levels of Parkin, PINK1, BNIP3L and LC3-II (p < 0.05). ARPE-19 cells pretreated with CsA under high glucose stress showed markedly down-regulated expressions of Parkin, PINK1 and BNIP3L compared with the cells treated with high glucose (p < 0.05). Pretreatment of ARPE-19 cells with NAC or 3-MA under high glucose stress resulted in a marked reduction in the expression levels of PINK1, BNIP3L and LC3-II (p < 0.05). Meanwhile, the expression level of Parkin in the ARPE-19 cells pretreated with NAC under high glucose stress was comparable with that in the control cells.

**Conclusion:**

Autophagy might have protective roles against high glucose induced injury in ARPE19 cells via regulating PINK1/Parkin pathway and BNIP3L.

## Background

The prevalence of diabetes is rising notably and the world prevalence of diabetes has been estimated to be 7.7%, affecting 439 million adults by 2030 [1, 2]. Diabetic retinopathy (DR), the most common visual complication of diabetes, is the leading cause of blindness among working-aged adults worldwide [3]. Previous individual studies have shown that the overall prevalence of DR was approximately 35% according to the clinical data from individuals with diabetes [4, 5].

DR is mainly caused by hyperglycemia, hypertension, dyslipidemia and defects of insulin signaling pathways [6, 7]. Autophagy is a lysosomal degradation pathway that controls cellular bioenergetics and cytoplasmic quality [8]. Autophagy is regulated by multiple forms of cellular stress, such as endoplasmic reticulum (ER) stress, nutrient or growth factor deprivation, hypoxia, cytotoxicity and infection [9, 10]. Mitophagy is a specialized process of autophagy that could reduce cellular damage and eliminate the need to support ineffective organelles by removing damaged mitochondria where increased levels of reactive oxygen species (ROS) are generated [11]. Accumulating data have suggested that autophagy have important effects on the pathobiology of DR [7, 12, 13].

Autophagy could protect against senescence and apoptosis of endothelial cells via renin–angiotensin system–mitochondrial damage induced by high glucose [14]. The microtubule-associated protein 1 light chain 3 (LC3) including cytosolic form (LC3-I) and membrane-bound lipidated form (LC3-II) has been commonly used to monitor autophagy [15]. It has been reported that Parkin (an E3 ubiquitin-protein ligase encoded by the *PARK2* gene) is selectively recruited to dysfunctional mitochondria by phosphatase and tensin homolog (PTEN)-induced putative kinase 1 (PINK1) in mammalian cells and in human fibroblasts treated with mitochondrial uncoupler carbonyl cyanide m-chlorophenylhydrazone (CCCP, mitophagy activator) [16, 17]. Bcl-2/adenovirus E1B 19 kDa-interacting protein 3-like protein (BNIP3L, also known as Nix) acted as a mitochondrial stress sensor can regulate mitophagy by promoting the release of Beclin1 from the Beclin1-Bcl2/Bcl-X complex [18, 19]. Therefore, the LC3-I, LC3-II, Parkin, PINK1 and BNIP3L might play important roles in autophagy.

The retinal pigment epithelium (RPE) are the main cells of the retina that situate between the neural retina and the choriocapillaris forming the outer blood–retinal barrier (BRB), and RPE cells also play important roles in the physiopathology of DR [20]. Studies have shown that ER stress markers, phospho-eIF2α and C/EBP homologous protein (CHOP), were significantly up-regulated in human retinal capillary endothelial cells and retinal pigment epithelial (RPE) cells cultured under high glucose condition, and in the retina of diabetic animal models [21–23]. Metabolic changes cause the RPE cells disorder, which lead to microvascular leakage in diabetes [24]. High glucose could contribute to the cell migration of RPE cells through increased oxidative stress and pigment epithelium-derived factor (PEDF) expression [25]. However, the molecular mechanisms of autophagy in the pathogenesis of DR still remain elusive. In this present study, we aimed to investigate the role of autophagy machinery in modulating RPE (ARPE-19 cells) under high glucose stress.

## Methods

### Cell culture

Human retinal pigment epithelium cell line, ARPE-19, was purchased from the American Type Culture Collection (ATCC, Mantissa, VA, USA). The ARPE-19 cells were maintained in Dulbecco’s Modified Eagle Medium mixed with Ham’s F-12 medium (DMEM/F12, Gibco, Grand Island, USA). Meanwhile, the medium was supplemented with 10% (v/v) heat-inactivated fetal bovine serum (FBS, Hyclone, Logan, UT), 100 units/ml of penicillin and 100 mg/ml of streptomycin. All the ARPE-19 cells were cultured in a humidified incubator of 5% CO_2_ at 37 °C. The ARPE-19 cells at exponential growth phase were digested with 0.25% trypsin for inoculation.

### Cell treatment and cell viability assay

ARPE-19 cells were seeded at a concentration of 24,000 cells/well in 6 well culture dishes until reaching 70–80% confluent, and the serum-free DMEM/F-12 was added to the cells for 24 h. The solutions of mitophagy activator carbonyl cyanide m-chlorophenylhydrazone (CCCP; Abcanm, Cambridge, MA), autophagy inhibitor 3-methyladenine (3-MA; Sigma, USA), ROS scavenger *N*-acetyl cysteine (NAC; Sigma, USA) or mitophagy inhibitor cyclosporin A (CsA; Sigma, USA) were prepared. Then, cells were incubated with d-glucose (5.5 mM [control], 30, 50 or 70 mM; Sigma, USA), NAC (0 [control], 1, 2 or 4 mM), CCCP (0 [control], 10, 20 or 30 mM;), CsA (0 [control], 5, 10 or 20 mM; Sigma, USA) or 3-MA (0 [control], 5, or 10 mM). The cell viability was measured after incubated at 37 °C in a humidified incubator of 5% CO_2_ for 48 h.

The concentrations of glucose, NAC, CCCP, CsA and 3-MA were selected according to the cell viability. ARPE-19 cells were seeded and incubated with 5.5 mM glucose (control group), high glucose (HG group) or the selected concentrations of small molecular drugs (NAC, CCCP, CsA or 3-MA) for 1 h. Afterwards, the high glucose solution was added to cells for an additional incubation for 48 h.

Cell viability was measured using a Cell Counting Kit-8 (CCK-8, Roche, Mannheim, Germany). After the indicated treatments, the cells were washed and CCK-8 solution (10 μl, 1:100 dilution) was added to each well, followed by incubation for 3 h at 37 °C. The absorbance at the wavelength of 450 nm was determined by a microplate reader (Tecan, Mechelen, Belgium). Cell viability was calculated according to the following formula: cell viability (%) = (OD treatment/OD control) × 100%.

### Morphological changes

After exposure to high glucose stress, morphological changes of ARPE-19 cells were detected using the transmission electron microscopy. ARPE-19 cells were fixed with 2.5% glutaraldehyde in phosphate buffered saline (PBS, pH 7.4) overnight, and post-fixed with 1% osmium tetroxide in 0.1 M cacodylate buffer for another 1 h. Samples were dehydrated with a graded series of ethanol solutions and embedded in araldite resin. Ultrathin sections (50–70 nm) were prepared using an ultramicrotome (Leica, Germany), stained with uranylacetate and lead citrate, and then scanned by transmission electron microscopy (Hitachi H-7500, Japan) at 80 kV. The cellular damage under high glucose stress was compared with that in the control group.

### Examination of intracellular ROS generation

The intracellular ROS generation was measured using the fluorescent probe 2,7-dichlorodihydrofluorescein diacetate oxidation (DCFH-DA, Sigma, USA) by flow cytometry [26]. Briefly, ARPE-19 cells were incubated with 10 mM DCFH-DA for 30 min at 37 °C in the dark. Cells were then washed with PBS and resuspended in PBS at a density of 1 × 10^6^ cells/ml. The untreated cells were served as the control. The levels of ROS were analyzed using flow cytometry at the excitation and emission wavelengths of 488 and 525 nm, respectively.

### Western blot analysis

The total proteins were extracted from cells by Super RIPA Lysis Buffer with Benzonase Nuclease (HaiGene, Harbin, China). The protein concentration was determined using the bicinchoninic acid (BCA) assay. Then, 30 μg of extracted protein was electrophoresed on the 8–12% sodium dodecyl sulfate polyacrylamide gel electrophoresis (SDS-PAGE) gel at 150 V for 2 h. After electrophoresis, the proteins were transferred onto nitrocellulose membranes (PALL, Mexico) at 100 mA for 2 h. The membranes were blocked in Tris-buffered saline (TBS) containing 5% skim milk powder for 1 h. After blocking, the membranes were incubated overnight at 4 °C with the primary antibodies of Parkin (1:1000; Abcam, Cambridge, UK), PINK1 (1:1000; Abcam, Cambridge, UK), BNIP3L (1:1000; Abcam, Cambridge, UK), LC3 (1:1000; CST, Boston, USA) and β-actin (1:2000; Genscript, Nanjing, China) at the recommended dilution. The membranes were then washed with TBS containing Tween 20 (TBST) and incubated with secondary antibodies (1:5000; HaiGene, Harbin, China) for 30 min at room temperature. The formed immunocomplex was visualized by enhanced chemiluminescence reagent (ECL; HaiGene, Harbin, China) and scanned by LAS-4000 Imaging System (FujiFilm, USA). The data were quantified using Image Pro-Plus and normalized by β-actin in triplicate.

### Statistical analysis

All measurement data were expressed as mean ± standard deviation (SD) and tested for normality of distribution using the Shapiro–Wilk test. Differences among multiple groups were analyzed by one-way analysis of variance (ANOVA) using SPSS version 13.0 software (SPSS, Inc., Chicago, IL, USA), followed by the Student–Newman–Keuls test. A value of p < 0.05 was considered to be statistically significant.

## Results

### High glucose treatment

**Fig. 1:**
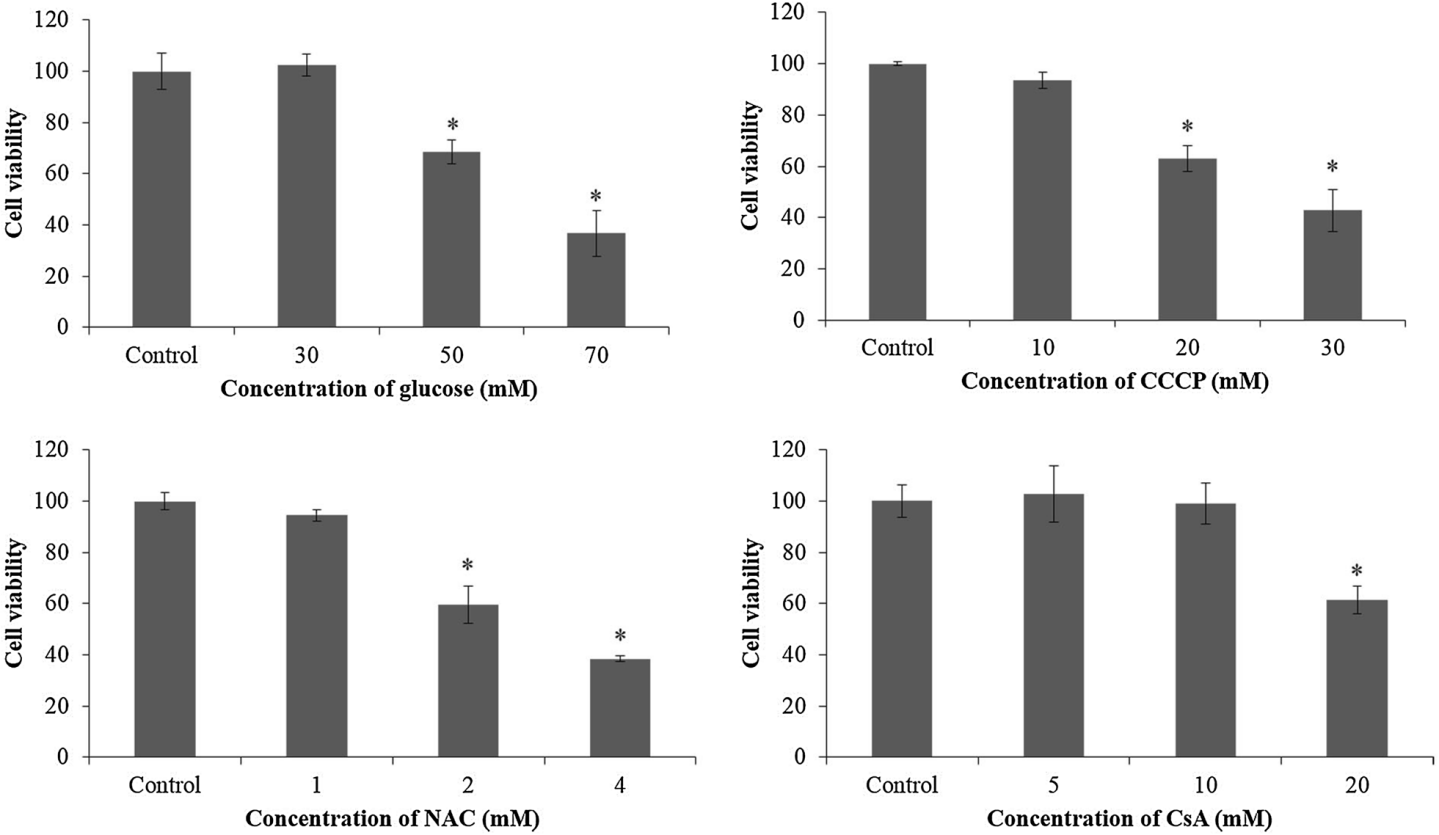
The viability of ARPE-19 cells exposured to different experimental conditions. The ARPE-19 cells were incubated with d-glucose (5.5 mM [control], 30, 50 or 70 mM), CCCP (0 [control], 10, 20 or 30 mM), NAC (0 [control], 1, 2 or 4 mM), or CsA (0 [control], 5, 10 or 20 mM) for 48 h (*p < 0.05 vs control)

After the cells were exposured to high glucose for 48 h, the ARPE-19 cells treated with 50 mM (68.48 ± 4.57%) or 70 mM (36.69 ± 8.94%) d-glucose had significantly lower cell viability (p < 0.05). However, there was no significant difference in the cell viability between the cells treated with 30 mM d-glucose and the control cells (102.33 ± 4.23% vs 99.99 ± 7.05%; p > 0.05; Fig. 1). Therefore, the ARPE-19 cells were subjected to 30 mM d-glucose for high glucose treatment in this study.

Meanwhile, the viability of ARPE-19 cells incubated with 10 mM of CCCP (93.56 ± 3.00%), 1 mM of NAC (94.51 ± 2.19%), 10 mM of CsA (98.91 ± 7.99%) or 5 mM of 3-MA did not decrease significantly as compared to the control cells (p > 0.05; Fig. 1). In our following study, the ARPE-19 cells were pretreated with NAC (1 mM), CCCP (10 mM), CsA (10 mM) or 3-MA (5 mM) for 1 h before incubated with 30 mM high glucose for 48 h.

### Structural changes and ROS generation induced by high glucose

**Fig. 2:**
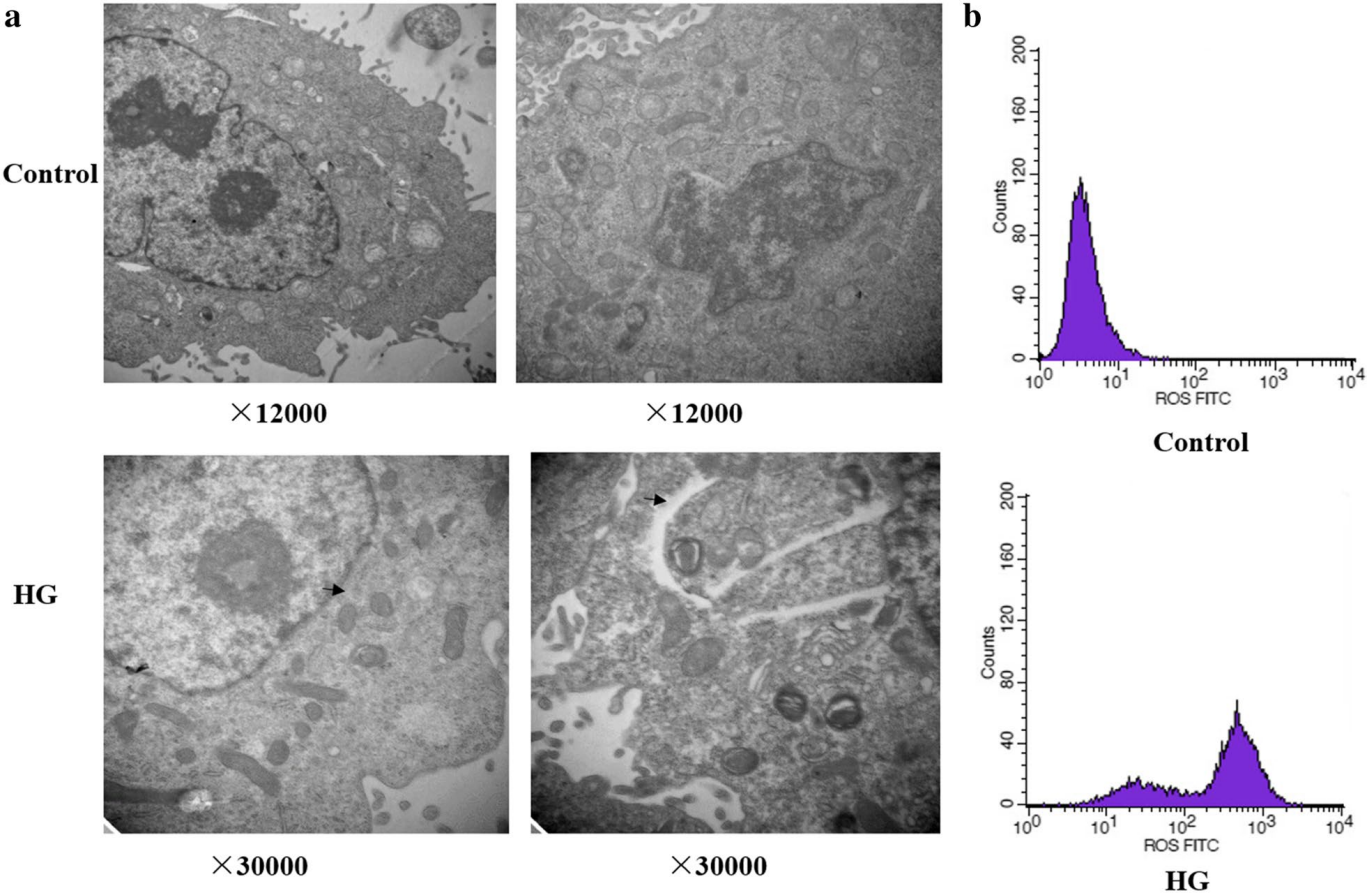
Intracellular structure changes and generation of reactive oxygen species induced by high glucose. The transmission electron microscopy (**a**) and flow cytometry analysis (**b**) of ARPE-19 cells treated with normal glucose (5.5 mM; Control; ×12,000) or high glucose (30 mM; HG; ×30,000) for 48 h. The autophagosomes in ARPE-19 cells were indicated by arrows

The transmission electron microscopy revealed that the ARPE-19 cells incubated with normal glucose (5.5 mM; control) or high glucose (30 mM; HG) for 48 h had a significant increase in the number of double-membrane vacuoles, which were typical of autophagosomes (Fig. 2a). Meanwhile, the levels of intracellular ROS in the ARPE-19 cells under high glucose stress were markedly higher in comparison with those of the control cells (Fig. 2b).

### Pretreatment with CCCP under high glucose stress

**Fig. 3:**
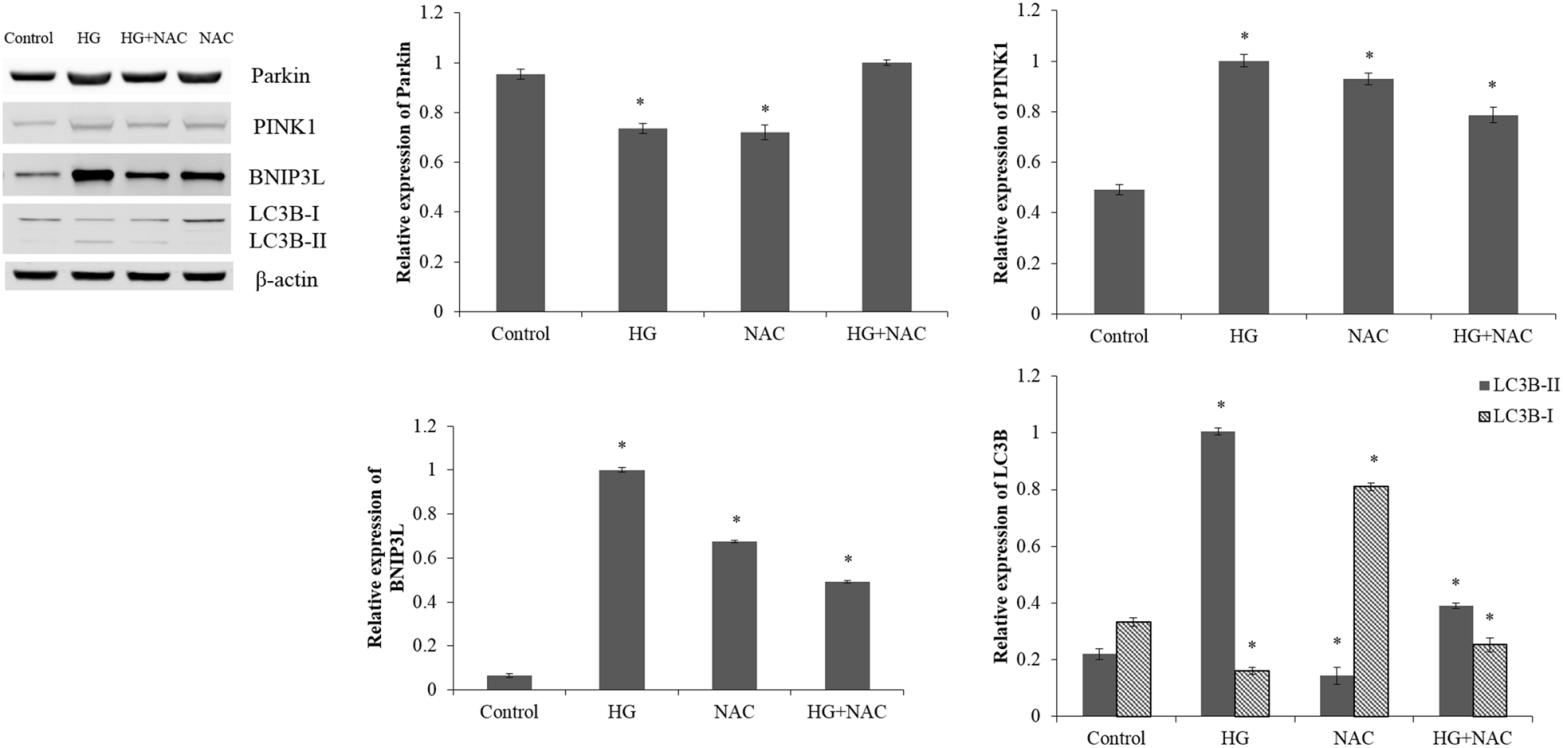
The expression levels of Parkin, PINK1, BNIP3L, LC3-I and LC3-II in the ARPE-19 cells treated with normal glucose (5.5 mM; Control) or high glucose (30 mM; HG) for 48 h; pretreated with CCCP (10 mM) for 1 h and then exposed to normal glucose (5.5 mM; 3-MA) or high glucose (30 mM; HG + 3-MA) condition. *p < 0.05 vs control

The expression levels of Parkin, PINK1, BNIP3L and LC3-II in the ARPE-19 cells under high glucose stress were significantly higher than those in the control cells (p < 0.05; Fig. 3). Meanwhile, the LC3-I level of ARPE-19 cells cultured under high glucose stress was obviously reduced, comparing with the control cells (p < 0.05).

After the ARPE-19 cells were pretreated with CCCP, the expressions of Parkin, PINK1, BNIP3L and LC3-II were significantly elevated in cells under high glucose stress (p < 0.05; Fig. 3). Moreover, the expression of LC3-I was obviously lower in the cells pretreated with CCCP under high glucose stress than the cells under high glucose stress without pretreatment (p < 0.05).

### Pretreatment with CsA, 3-MA or NAC under high glucose stress

**Fig. 4:**
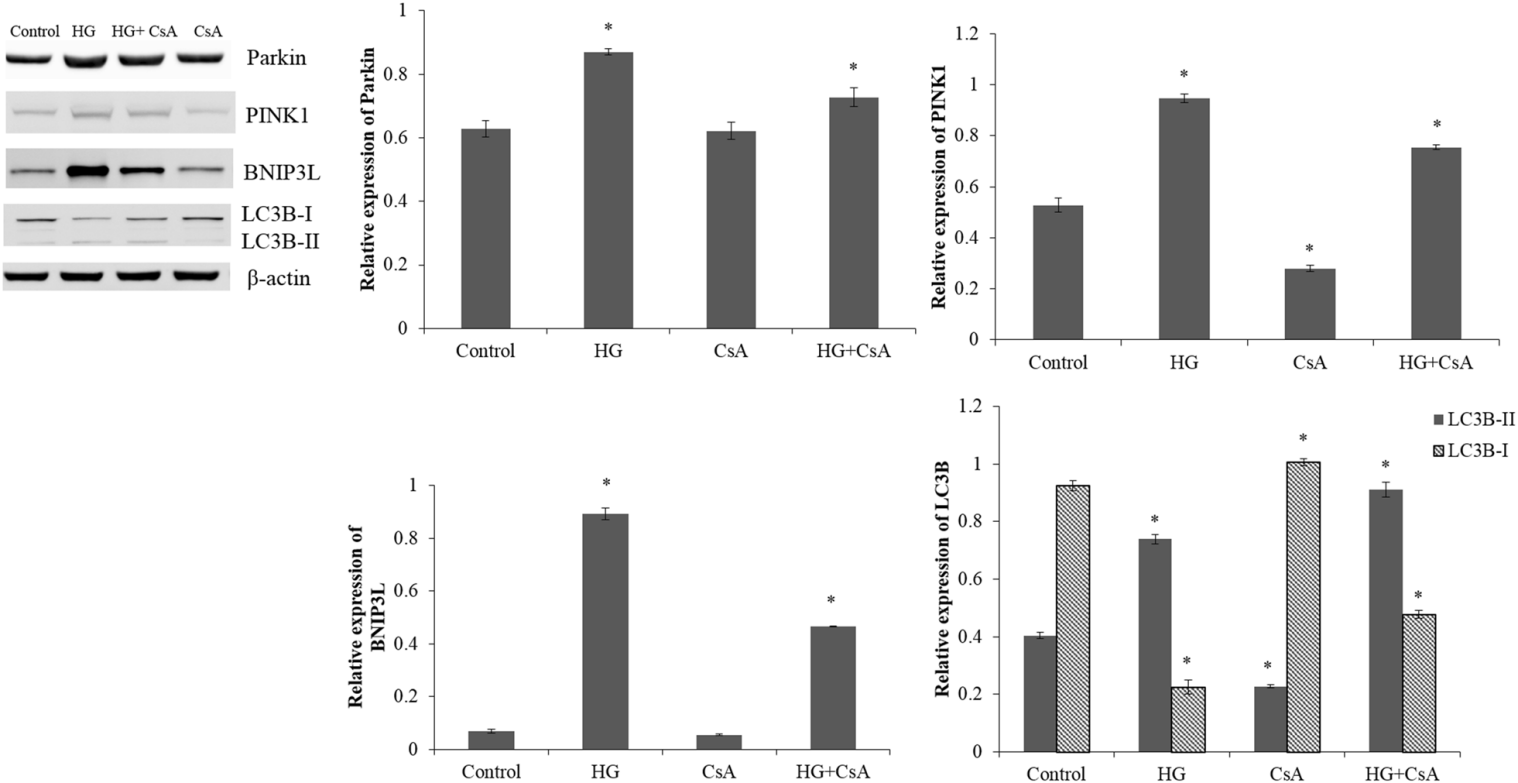
The western blot analysis of ARPE-19 cells in normal glucose (5.5 mM; Control) or high glucose (30 mM; HG) condition; pretreated with CsA (10 mM) for 1 h and then exposed to normal glucose (5.5 mM; CsA) or high glucose (30 mM; HG + CsA) for 48 h. *p < 0.05 vs control

**Fig. 5:**
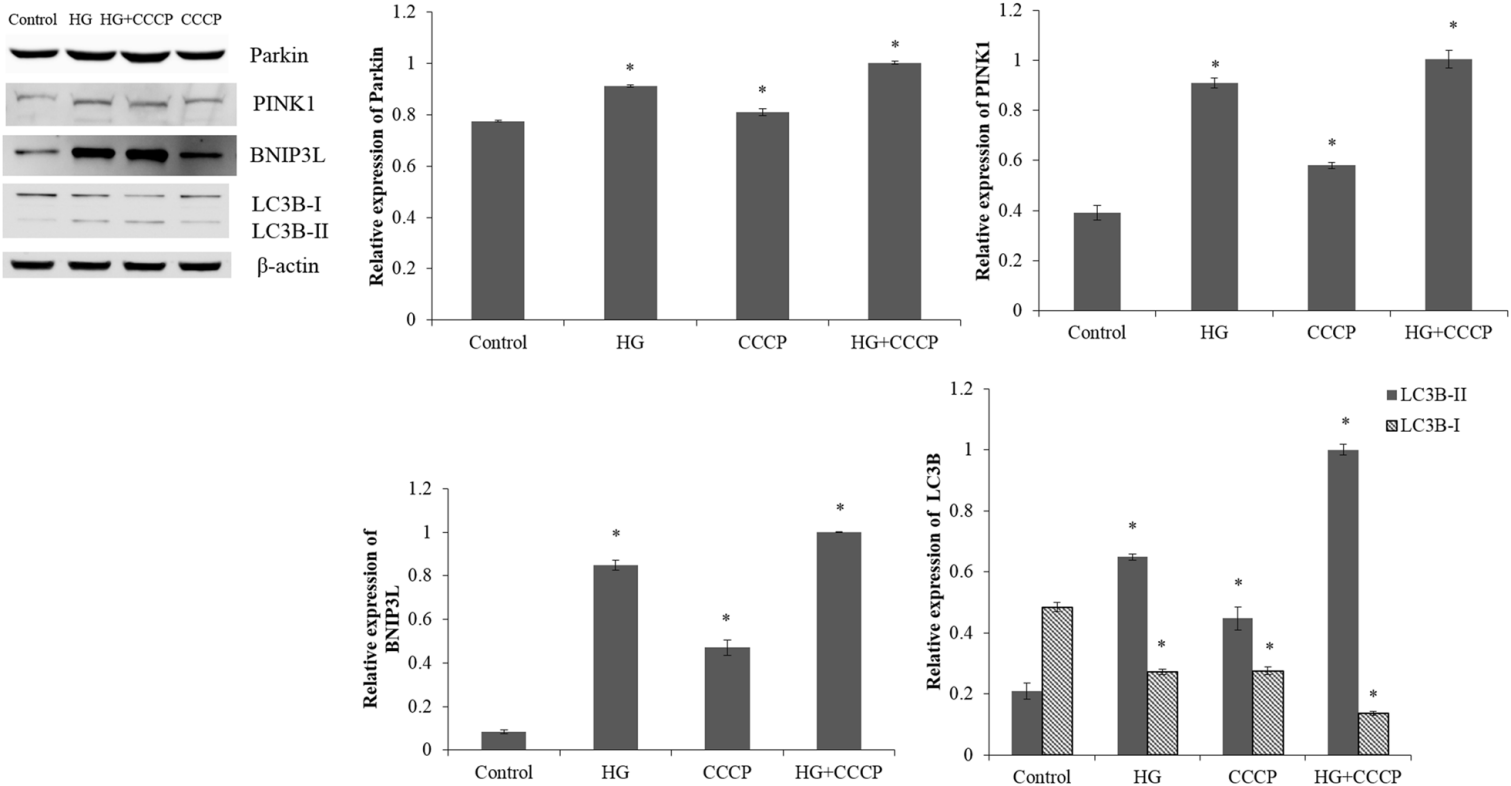
The expression levels of Parkin, PINK1, BNIP3L, LC3-I and LC3-II in the control cells, HG cells, cells pretreated with 5 mM 3-MA and then exposed to normal glucose (5.5 mM; 3-MA) or high glucose (30 mM; HG + 3-MA). *p < 0.05 vs control

Compared with the ARPE-19 cells exposed to normal glucose, high glucose treatment caused a significant increase in the expression levels of Parkin, PINK1, BNIP3L and LC3-II (p < 0.05; Figs. 4, 5). Meanwhile, high glucose stress leaded to a reduction in LC3-I expression in ARPE-19 cells compared with the control cells (p < 0.05).

**Fig. 6:**
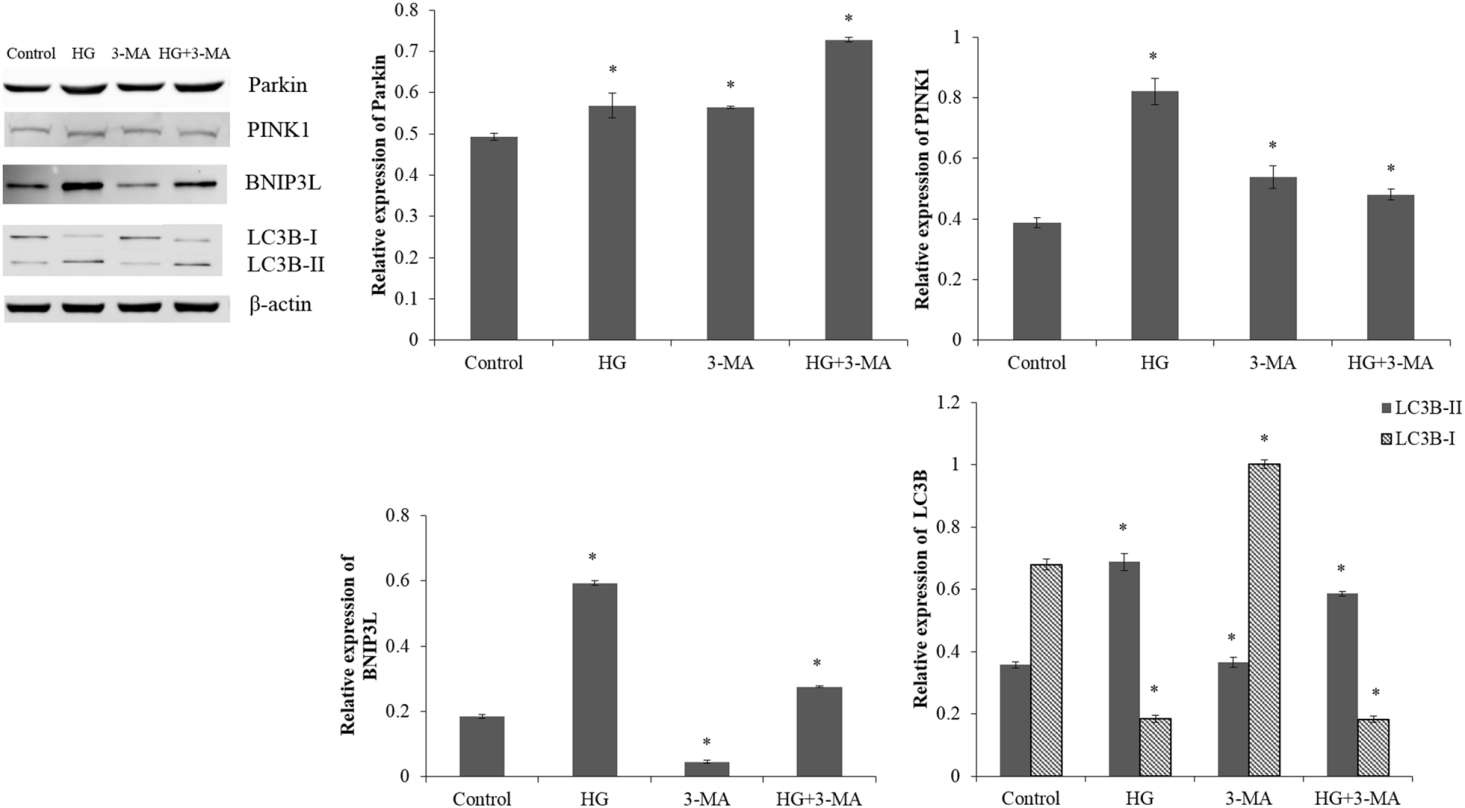
The western blot analysis of Parkin, PINK1, BNIP3L LC3-I and LC3-II in the control cells, HG cells, cells pretreated with NAC (1 mM) and then exposed to normal glucose (5.5 mM; NAC) or high glucose (30 mM; HG + NAC). *p < 0.05 vs control

The ARPE-19 cells pretreated with CsA under high glucose stress showed markedly down-regulated expressions of Parkin, PINK1 and BNIP3L compared with the cells under high glucose stress without pretreatment (p < 0.05; Fig. 4). The CsA, an inhibitor of the mitochondrial permeability transition pore [27], might blocked the high glucose induced mitophagy and PINK1/Parkin pathway. The ARPE-19 cells pretreated with 3-MA under high glucose stress showed markedly up-regulated expression of Parkin and down-regulated expressions of PINK1, BNIP3L and LC3-II compared with the cells under high glucose stress without pretreatment (p < 0.05; Fig. 5). After the ARPE-19 cells were pretreated with NAC, the expression levels of PINK1, BNIP3L and LC3-II were significantly reduced under high glucose stress (p < 0.05; Fig. 6). The ARPE-19 cells treated with high glucose or NAC showed an obvious reduction in the Parkin expression (p < 0.05), while no significant difference was observed in Parkin expression between the ARPE-19 cells treated with NAC under high glucose stress and control cells (p > 0.05; Fig. 6).

## Discussion

Hyperglycemia which is responsible for the initiation and progression of DR is a serious complication of diabetes mellitus [28]. In the present study, the number of autophagosomes, intracellular ROS level and expression of LC3-II were markedly increased in the ARPE-19 cells treated with high glucose. Meanwhile, ARPE-19 cells showed an obvious reduction in the LC3-I levels under high glucose stress. LC3-II is formed via phosphatidylethanolamine conjugation of LC3-I representing on isolation membranes and autophagosomes [29]. Detecting the conversion of LC3-I to LC3-II is used for monitoring the number of autophagosomes and autophagic flux [30]. Therefore, our data suggested that high glucose caused the autophagy in ARPE-19 cells. In our further study, the ARPE-19 cells will be treated by Bafilomycin A1, an inhibitor of lysosomal degradation, to further interpret the decreased amounts of LC3-I. The ARPE-19 cells treated with high glucose showed markedly increased generation of autophagosome, down-regulated expression of p62 and enhanced LC3-II conversion [31]. The expression levels of p62/SQSTM1 or optineurin will be measured to further interpret the increase in LC3-II and autophagosomes in our next study.

Hyperglycemia induced oxidative stress is implicated in the development of DR, and it has been clarified that overexpression of manganese superoxide dismutase (MnSOD) in endothelium could prevent DR according to the study of diabetic transgenic mice [32]. In our study, the intracellular ROS levels in ARPE-19 cells were significantly elevated in response to high glucose stress. The expressions of PINK1 and BNIP3L were also obviously increased in the ARPE-19 cells under high glucose stress comparing with the control cells. It has been reported that BNIP3L could promote CCCP-induced mitochondrial depolarization and ROS generation to inhibit rapamycin (mTOR) signaling and activate autophagy [33]. In our study, down-regulated expression of LC3-I and up-regulated expression levels of Parkin, PINK1, BNIP3L and LC3-II were observed in RPE-19 cells pretreated with mitophagy activator CCCP under high glucose stress. The rapid accumulation of PINK1 on the outer mitochondrial membranes caused by polarized mitochondria (loss of mitochondrial membrane potential) can lead to the translocation of Parkin to mitochondria [34, 35]. Then, autophagic degradation of the dysfunctional mitochondrion caused by the recruited Parkin ubiquitinating mitochondrial proteins in the outer membrane indicates PINK1 acts as upstream of Parkin to regulate mitochondrial function and integrity [36, 37]. Therefore, it could be concluded that CCCP might activate the mitophagy in ARPE-19 cells via up-regulating the Parkin, PINK1 and BNIP3L. However, this should be further validated by immunofluorescence for o-localization of mitochondrial ETC complex protein COXIV (cytochrome c oxidase, subunit IV, a mitochondrial marker) with Parkin and Lamp 2a.

Inhibition of autophagy in R28 retinal neuronal cells with chloroquine significantly exacerbated the expressions of apoptotic proteins and apoptotic cell death during metabolic stress, suggesting autophagy modulation might be a novel therapeutic strategy for the prevention of neurodegeneration in DR [38]. Cyclosporin A, an immunosuppressive undecapeptide, blocks the mitochondrial permeability transition (MPT) and prevents mitochondrial depolarization after autophagic stimulation [39]. In our study, the ARPE-19 cells pretreated with CsA under high glucose stress showed markedly down-regulated expressions of Parkin, PINK1 and BNIP3L compared with the cells under high glucose stress without pretreatment. We also found that pretreatment of ARPE-19 cells with NAC under high glucose stress could result in obviously reduced expression levels of PINK1, BNIP3L and LC3-II. Meanwhile, the Parkin expression level in the ARPE-19 cells treated with NAC under high glucose stress was comparable with that in the control cells. Endoplasmic reticulum stress can be activated by high glucose induced accumulation of ROS, and autophagy regulated by ER stress is critical for the maintenance of normal physiological functions in retinal pigment epithelium [31]. Thus, interruption of ER stress signaling via NAC to scavenge ROS can lead to the inhibition of autophagy in ARPE-19 cells. In the study of Du et al. [40], the generation of ROS was increased in the retina vascular endothelial cells under the high glucose stress and decreased in the cells under high glucose stress pretreated with NAC. Increased autophagy has been observed in ARPE-19 cells under high glucose stress, and inhibition of autophagy by 3-MA (a type III PI3 kinase complex inhibitor) could induce interleukin (IL)-1β release via ROS mediated pyrin domain-containing 3 (NLRP3) inflammasome [24]. In our study, suppression of autophagy in ARPE-19 cells under high glucose stress by 3-MA could cause a marked reduction in the expression levels of PINK1, BNIP3L and LC3-II.

## Conclusion

In conclusion, our study showed autophagy might have protective roles against high glucose induced injury in ARPE19 cells via regulating PINK1/Parkin pathway and BNIP3L. The autophagy in ARPE-19 cells was enhanced under high glucose stress. Activation of autophagy by pretreatment of CCCP increased the expression levels of Parkin, PINK1 and BNIP3L. Inhibition of autophagy by pretreatment of CsA, NAC or 3-MA under high glucose stress caused markedly down-regulated expressions of Parkin, PINK1 and BNIP3L. Our present study might be helpful for understanding the molecular mechanisms of DR.
